# Intercalation and Push‐Out Process with Spinel‐to‐Rocksalt Transition on Mg Insertion into Spinel Oxides in Magnesium Batteries

**DOI:** 10.1002/advs.201500072

**Published:** 2015-06-10

**Authors:** Shinya Okamoto, Tetsu Ichitsubo, Tomoya Kawaguchi, Yu Kumagai, Fumiyasu Oba, Shunsuke Yagi, Kohei Shimokawa, Natsumi Goto, Takayuki Doi, Eiichiro Matsubara

**Affiliations:** ^1^ Department of Materials Science and EngineeringKyoto UniversityKyoto606‐8501Japan; ^2^ Materials Research Center for Element StrategyTokyo Institute of TechnologyYokohama226‐8503Japan; ^3^ Nanoscience and Nanotechnology Research CenterOsaka Prefecture UniversityOsaka599‐8570Japan; ^4^ Department of Molecular Chemistry and BiochemistryDoshisha UniversityKyoto610‐0321Japan

## Abstract

On the basis of the similarity between spinel and rocksalt structures, it is shown that some spinel oxides (e.g., MgCo_2_O_4_, etc) can be cathode materials for Mg rechargeable batteries around 150 °C. The Mg insertion into spinel lattices occurs via “intercalation and push‐out” process to form a rocksalt phase in the spinel mother phase. For example, by utilizing the valence change from Co(III) to Co(II) in MgCo_2_O_4_, Mg insertion occurs at a considerably high potential of about 2.9 V vs. Mg^2+^/Mg, and similarly it occurs around 2.3 V vs. Mg^2+^/Mg with the valence change from Mn(III) to Mn(II) in MgMn_2_O_4_, being comparable to the ab initio calculation. The feasibility of Mg insertion would depend on the phase stability of the counterpart rocksalt XO of MgO in Mg_2_X_2_O_4_ or MgX_3_O_4_ (X = Co, Fe, Mn, and Cr). In addition, the normal spinel MgMn_2_O_4_ and MgCr_2_O_4_ can be demagnesiated to some extent owing to the robust host structure of Mg_1−x_X_2_O_4_, where the Mg extraction/insertion potentials for MgMn_2_O_4_ and MgCr_2_O_4_ are both about 3.4 V vs. Mg^2+^/Mg. Especially, the former “intercalation and push‐out” process would provide a safe and stable design of cathode materials for polyvalent cations.

## 1 Introduction

In terms of energy and environmental concerns, modern industrial society strongly demands high energy‐density rechargeable storage batteries. Currently, lithium ion batteries (LIBs) are widely used for a lot of practical applications, and their energy density has been enlarged year by year, but its growing rate tends to be saturated recently. If lithium metal itself could be used as an anode material instead of carbonaceous materials currently used, LIBs would have shown significantly high energy densities, but this cannot be done due to the well‐known fatal problem, “dendritic growth” of Li metal on charge that leads to dangerous short circuits.^1^ Therefore, in order to further enhance the energy density of storage batteries, we have to develop new type of metal‐anode battery systems.

As an alternative to Li metal‐anode battery, polyvalent‐metal (Mg, Ca, Al, etc) storage batteries (PSBs) have recently attracted increased attention owing to their large capacities; for example, in the case of Mg, its capacity (ca. 2200 mAh g^−1^) largely exceeds that for the current carbonaceous anode materials (ca. 370 mAh g^−1^). Especially, it has been reported that Mg electrodeposition occurs with non‐dendritic formation;^2^, ^3^, ^4^ therefore Mg metal can be expected to work as an anode material. Thus, the Mg rechargeable battery (MRB) field has been currently attracting much attention but growing up quite gradually. Namely, the MRB research is still a very challenging field and not established yet, and hence we have to make much effort to accomplish MRBs. For example, there are no appropriate electrolytes with wider electrochemical windows and without causing passivation on the Mg‐electrode surface. Furthermore, despite that several candidates for the MRB cathode materials have been reported,^5^, ^6^, ^7^ there are few cathode materials for MRBs that can work at ambient temperature except for Chevrel compounds;^8^, ^9^ even though the Chevrel compounds are used, the electromotive force delivers about 1.0–1.2 V, and the energy density of Mg battery is less than 150 mWh g^−1^ (currently 370 mWh g^−1^ in electrode energy density of LiCoO_2_ vs graphite). Therefore, unless more talented cathode materials that can accommodate polyvalent cations are sought out, PSBs would not be comparable to Li ion batteries in terms of the energy density. Thus, in order to change the energy storage paradigm, we have to seek cathode materials for polyvalent cations.

**Figure 1:**
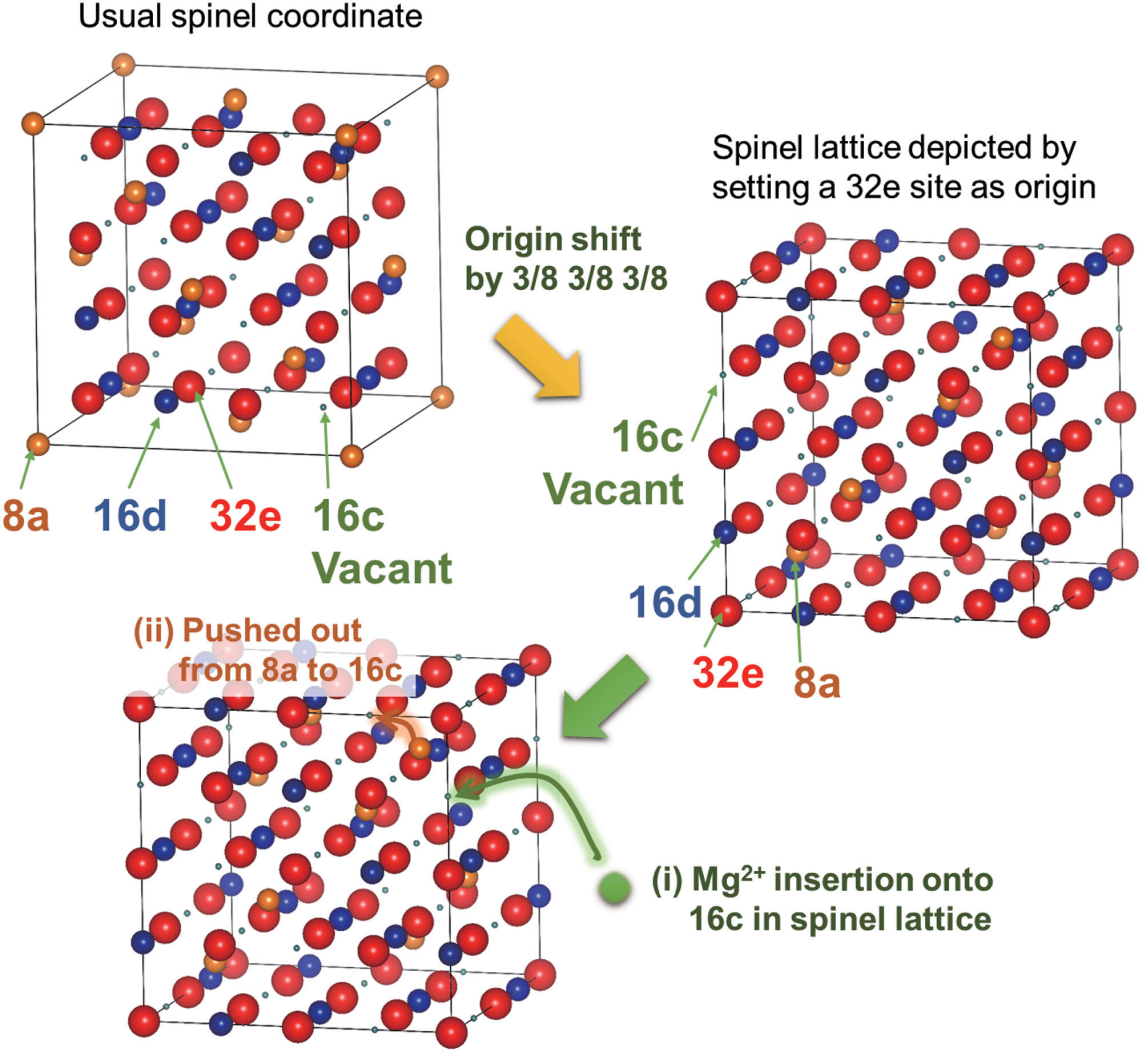
Schematic illustration showing the cation insertion process. The upper left structure is drawn in the usual spinel coordinate, whereas the right is depicted by setting a 32e site (Wyckoff position) for oxygen in the space group No. 227 as origin. After a Mg cation is inserted into a 16c site in the spinel (left lower) structure, the original cation located in its neighboring 8a site moves to an adjacent 16c site due to the repulsion between the cations.

Here we focus Mg spinel oxides as candidates for cathode materials of MRBs. As shown in **Figure** **1**, the lattice sites in the spinel structure are generally denoted as 8a, 16d (cation sites), and 32e (oxygen sites) in the Wyckoff position in the space group No. 227 ($Fd\bar{3}m$), while those in the rocksalt structure are denoted as 16c, 16d (cation sites) and 32e (oxygen sites) when it is assigned to the same space group. Thus, a spinel structure can be regarded as a rocksalt whose 16c sites are vacant and instead the 8a sites are usually occupied by cations. Therefore, it is expected that Mg cations can be inserted onto 16c vacant sites in the spinel lattice, as well as the Li insertion mechanism in spinel oxide materials.^10^, ^11^

In this work, with several spinel oxides MgCo_2_O_4_, MgMn_2_O_4_, MgFe_2_O_4_, MgCr_2_O_4_, and Co_3_O_4_, we demonstrate that some of spinel oxides can allow the insertion of Mg cations at high potentials (about 3 V vs. Mg^2+^/Mg) via “intercalation and push‐out” mechanism as shown in Figure 1. The electrochemical‐test temperature was set at 150 °C in the present study by the following two reasons: i) the melting temperature (about 120 °C) of the CsTFSA based ionic liquids^12^, ^13^ used here and ii) the enhancement of Mg diffusion in the active materials. Actually, the future Mg battery is expected to be operated at moderately high temperatures in that Mg insertion and extraction can be facilitated at such temperatures. Finally, we discuss the feasibility of Mg insertion/extraction into/from the spinel oxides in terms of stabilities of the resultant rocksalt phases and the original spinel structure types.

## 2 Results and Discussion

### 2.1 Redox Behavior of Spinel Oxides

**Figure 2:**
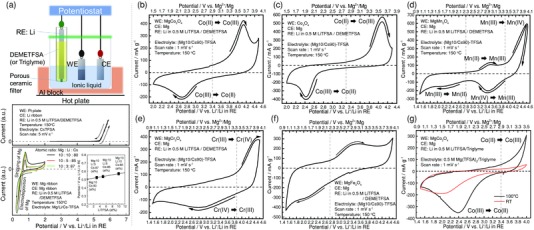
a) Three‐electrode beaker cell setup (upper): (Mg10/Cs90)‐TFSA ionic liquid was used for the electrolyte, the cathode active material was used as the working electrode (WE), a Mg ribbon was counter electrode (CE), and Li metal immersed in a 0.5 m‐LiTFSA/DEMETFSA electrolyte in a glass tube separated with a porous ceramic filter was used as the reference electrode (RE). Cyclic voltammogram (lower) measured at 150 °C in the ionic liquid of the mixture of Mg(TFSA)_2_, LiTFSA, and CsTFSA salts with various concentrations to extrapolate the virtual redox potential of Mg^2+^/Mg couple in the (Mg10/Cs90)‐TFSA ionic liquid. The anodic limit of the electrochemical window was determined to be about 4.5 V vs. Li^+^/Li in RE by using the Cs‐TFSA ionic liquid (middle). Cyclic voltammograms measured at a scan rate of 1 mV s^−1^ at 150 °C for various spinel oxides; b) MgCo_2_O_4_, c) Co_3_O_4_, d) MgMn_2_O_4_, e) MgCr_2_O_4_, f) MgFe_2_O_4_, and g) MgCo_2_O_4_ in a 0.5 m Mg(TFSA)_2_/triglyme electrolyte at room temperature (RT) and 100 °C. All the voltammograms are of the 1st cycle except for MgCr_2_O_4_. For MgCr_2_O_4_, the voltammogram at the 2nd cycle is shown to eliminate the effect of the slight contamination with Na^+^ ions, which was confirmed by EDX elemental analysis.

A typical construction of beaker cells used here is illustrated in **Figure** **2**a (upper). In order to perform electrochemical tests around 150 °C, we used the CsTFSA‐based ionic liquids reported by Hagiwara et al.,^13^ which show excellent thermal stabilities around 200 °C. The electrodeposition of Mg hardly occurs in a Mg(TFSA)_2_/CsTFSA binary ionic liquid, but the electrolytic dissociation of Mg(TFSA)_2_ in the ionic liquid takes place, which is judged from the fact that the Mg cations can be inserted into the Chevrel compounds.^14^ Besides, as demonstrated in the previous works,^13^, ^15^ hcp Mg metal can be electrodeposited in (Mg/Li/Cs)‐TFSA ternary ionic liquids. Based on this fact, the virtual Mg redox potential is deduced by reducing the Li composition from the ternary ionic liquids. Thus, the potential conversion rule from the potential versus the reference electrode (RE) used in the present work to that versus Mg^2+^/Mg was tentatively determined to be “0.5 V vs. Li^+^/Li in RE ≈ 0 V vs. Mg^2+^/Mg”, as shown in Figure 2a (lower). Although, in addition to the electrodeposition/stripping phenomenon of Mg, the Li insertion into Mg metal matrix can be involved in the CV profiles, we have judged that the obtained CV profile shape is of typical electrodeposition/stripping pheno­menon; see SI for details. Incidentally, the anodic limit in the electrochemical window of the CsTFSA ionic liquid is about/below 4.5 V vs. Li^+^/Li in RE; see Figure S1, Supporting Information, for details.

Figure 2b shows the cyclic voltammogram (CV) obtained for MgCo_2_O_4_. Usually, one would consider the conventional reaction, i.e., the reaction of Mg extraction from the host material, MgCo_2_O_4_ ⇔ Mg_1−x_Co_2_O_4_ +*x*(Mg^2+^ + 2e^−^). However, such a reaction may occur above 4.4 V vs. Li^+^/Li in RE, but this cation extraction from MgCo_2_O_4_ appears to be difficult in terms of the structural stability and anodic limit of the electrochemical window of the binary ionic liquid. Apart from this usual deintercalation, it is clearly seen that direct Mg insertion can occur into the host MgCo_2_O_4_ without a pre‐charge process, and then the cation extraction is observed during a charge process; the equilibrium redox potential is about 2.9 V vs. Mg^2+^/Mg (3.4 V vs. Li^+^/Li in RE), which is in agreement with the ab initio calculation (3.0 V vs. Mg^2+^/Mg); see the later section on the ab initio calculation. Thus, the insertion/extraction of Mg cations are found to be drastically facilitated by elevating temperature. After the electrochemical tests, we confirmed Mg insertion semi‐quantitatively by the energy‐dispersive X‐ray (EDX) spectroscopy analysis (not presented here), and also we have performed the inductively coupled plasma (ICP) analysis after the electrochemical performance test for the Mg‐Li rocking‐chair type dual‐salt battery.^16^

As candidates of cathode active materials for MRB systems, other spinel oxides, Co_3_O_4_, MgMn_2_O_4_, MgCr_2_O_4_, and MgFe_2_O_4_, were also investigated. Figures 2c–f show cyclic voltammograms for these spinel oxides measured in the (Mg10/Cs90)‐TFSA binary ionic liquid at 150 °C. The CV profile in Figure 2c of Co_3_O_4_ is very similar to that of MgCo_2_O_4_, where the extraction of Co(II) cations is not observed during the anodic scan from the open circuit potential (OCP). The elemental analysis by EDX suggested that Mg cations were inserted into the spinel Co_3_O_4_ (note presented here). In contrast to MgCo_2_O_4_, the extraction of Mg^2+^ ions from MgMn_2_O_4_ is observed in Figure 2d during the first anodic scan from the OCP value, and two redox‐peak couples corresponding to the insertion/extraction of Mg^2+^ ions are observed at around 3.4 V and 2.3 V vs. Mg^2+^/Mg; the former reaction would correspond to the valence change of Mn(IV) to Mn(III), whereas the latter would correspond to that of Mn(III) to Mn(II). According to the present ab initio calculations of the energy differences between MgMn_2_O_4_ and demagnesiated‐spinel Mn_2_O_4_ and between MgMn_2_O_4_ and magnesiated‐spinel (i.e., rocksalt) Mg_2_Mn_2_O_4_, the average redox potentials were estimated to be 2.9 V vs. Mg^2+^/Mg for the former and 1.8 V vs. Mg^2+^/Mg for the latter, being in fairly agreement with the experimental results.

As to the former reaction, the similar trend was observed in Figure 2e for MgCr_2_O_4_, that is, the valance change from Cr(IV) to Cr(III) was observed, but in contrast, the valence change of Cr(III) to Cr(II) was hardly observed in this case. In the case of MgFe_2_O_4_, as well as the above materials, we confirmed that MgFe_2_O_4_ can be used as a cathode material for MRBs, but marked redox peaks were not observed in Figure 2(f); as a trend, the current density for this material is considerably lower than those in the other spinels. However, the faint peaks probably correspond to the insertion and extraction of Mg cations into MgFe_2_O_4_; the insertion/extraction potential is about 2.7 V vs. Li^+^/Li in RE, being lower than the redox potential (about 3.4 V vs. Li^+^/Li) of Fe cations in the olivine LiFePO_4_. This is further supported by the XRD and XANES measurements in the next section.

In the above cyclic voltammetry tests, the temperature was set at 150 °C. Here, we show the CV profiles of MgCo_2_O_4_ at relatively lower temperatures, by using the Mg(TFSA)_2_/triglyme electrolyte, which was recently developed for MRBs.^17^ Since the electrolyte is in a liquid state at room temperature and the boiling point of triglyme is about 216 °C, we can conduct electrochemical tests in a relatively wide temperature range. Figure 2g shows the cyclic voltammograms measured for MgCo_2_O_4_ in a triglyme electrolyte containing 0.5 m Mg(TFSA)_2_ at room temperature (RT) and 100 °C. The insertion of Mg^2+^ ions into the spinel MgCo_2_O_4_ was observed in each case (60 mAh g^−1^ for RT and 105 mAh g^−1^ for 100 °C) below 3.4 V vs. Li^+^/Li in RE. After the cathodic sweep to 1.5 V vs. Li^+^/Li in RE, the anodic current corresponding to the extraction of Mg^2+^ ions was markedly observed above 3.4 V vs. Li^+^/Li in RE, but it is found that the extraction of Mg^2+^ below 100 °C is considerably laborious within the electrochemical window of the triglyme. Thus, the triglyme electrolyte can be used for the evaluation of active materials at RT and/or higher temperatures.

### 2.2 Structure Analyses

**Figure 3:**
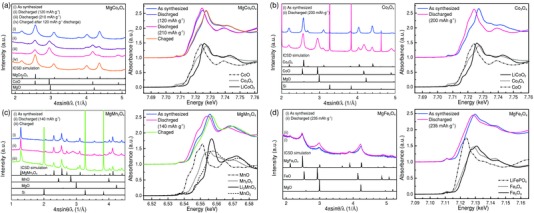
XRD profiles (left) and XANES spectra (right) measured for a) MgCo_2_O_4_, b) Co_3_O_4_, c) MgMn_2_O_4_, and d) MgFe_2_O_4_. The amount of discharge (Mg insertion) is denoted for each sample, but the charge amount is not denoted because the oxidation decomposition of the electrolyte is also included in the charge amount (over 4 V vs. Li^+^/Li in RE).

We conducted structural analyses for mainly MgCo_2_O_4_ before/after Mg insertion/extraction tests to comprehend the cation‐insertion mechanism. The analyses have been done for MgCo_2_O_4_, Co_3_O_4_, MgMn_2_O_4_, and MgFe_2_O_4_. As shown in XRD profiles in **Figure** **3**a (left), after insertion of Mg cations by discharge of about 120 mAh g^−1^, the active material contains two phases, i.e., spinel and rocksalt phases. Further insertion of Mg cations up to about 210 mAh g^−1^ substantially forms a rocksalt single phase. The fact that the spinel phase disappears even at such an incomplete discharge amount less than the theoretical value (260 mAh g^−1^) means that the rocksalt phase includes a certain amount of cation vacancies, that is, a solid‐solution phase of off‐stoichiometry exists. When charging after the discharge of 120 mAh g^−1^, the structure completely reverts to the spinel structure. By measuring the corresponding XANES spectra around the Co K‐edge in Figure 3a (right), we further ensure that a part of Co(III) cations in the spinel phase are reduced to Co(II) after discharge of about 120 mAh g^−1^ and again oxidized to Co(III) after charge; compare to the XANES profiles of Co_3_O_4_ containing Co(II) and Co(III), CoO with only Co(II), and LiCoO_2_ with only Co(III).

As well as MgCo_2_O_4_, we have also conducted XRD and XANES measurements for Co_3_O_4_ in Figure 3b, MgMn_2_O_4_ in Figure 3c, and MgFe_2_O_4_ in Figure 3d. As is expected easily, the Mg insertion/extraction behavior into/from Co_3_O_4_ in Figure 3b is very similar to that of MgCo_2_O_4_. Incidentally, a certain amount of Si was mixed to gain a sufficient sample volume in the capillary for the measurements. Namely, by the insertion of Mg cations (200 mAh g^−1^), the rocksalt phase is formed in the spinel mother phase. Comparing to MgCo_2_O_4_, the spinel phase tends to remain in the case of such an incomplete discharge amount. On the other hand, we observe not only the Mg insertion into a pristine MgMn_2_O_4_ but also the Mg extraction from such a pristine MgMn_2_O_4_, as seen in Figure 3c. As to the insertion, broad XRD peaks coming from the rocksalt phase are also observed in this case, whereas several new peaks are detected after the Mg extraction from the host material. As is seen in corresponding XANES spectra (Mn K‐edge), the white line of as‐synthesized MgMn_2_O_4_ shifts to a lower energy after discharge and shifts to a higher energy after charge. These behaviors are consistent with those of the reference samples corresponding to the various valence states of Mn. Finally, although MgFe_2_O_4_ did not show an excellent cathode property in Figure 2f, we have investigated the structural and valence changes after Mg insertion. As found from Figure 3d, the XRD peak positions of MgFe_2_O_4_, FeO and MgO are very close, so that we cannot clearly see the peak shifts in the broad XRD profile of pristine MgFe_2_O_4_. However, after the Mg insertion, the XRD peaks tend to move to lower angles (probably due to the influence of FeO) and the white line (Fe K‐edge) tends to shift lower. As seen in the XANES profiles of Fe_2_O_3_ and Fe_3_O_4_, there is only a little change in the respective white lines, and the present XANES profile change after Mg insertion is rather similar to this trend, unlike the Fe K‐edge profile in the olivine LiFePO_4_ structure.

As a representative of spinel oxide cathode materials for MRBs, the crystal structure parameters were determined by Rietveld refinement with the program RIETAN‐FP^18^ using the XRD profiles of the as‐synthesized MgCo_2_O_4_ sample and one after partial discharge (120 mAh g^−1^) in the Mg battery system that corresponds to Figure 3a. We mentioned before that the rocksalt phase would have vacancies after discharge, but we here assume that the discharged rocksalt structure does not have any vacancies. The cation ratio of the spinel structure was fixed at Mg/Co = 1/2, and no constraint was imposed on the cation ratio in the rocksalt structure, i.e., the discharge amount (120 mAh g^−1^) was not taken into account for the Rietveld analysis. The fitting results and detailed parameters for crystal structures are shown in Figure S3 and Table S1, Supporting Information. From the Rietveld analysis, as‐synthesized MgCo_2_O_4_ takes a disordered spinel structure with a degree of disorder of about 0.43, being consistent with our previous works.^7^, ^19^ In contrast, the partially discharged sample contains spinel and rocksalt phases, and the degree of disorder is slightly changed to about 0.37, and the site occupancies of Mg and Co cations in the rocksalt structure are 0.49 and 0.51, respectively. The volume fraction was determined to be spinel (27%) and rocksalt (73%) structures, indicating that about 70% of the discharge process proceeds in terms of the present structure analysis. Therefore, considering the fact that the discharge amount was less than half of the full capacity (120 mAh g^−1^/260 mAh g^−1^), we need to consider the presence of vacancies in the rocksalt crystal. Thus, the insertion of one Mg atom induces the spinel to rocksalt transition in a larger region than one unit cell of the rocksalt structure.

From the present structure analysis, the Mg cations are inserted into 16c sites in the spinel structure, and the original cations located at the 8a sites of the spinel structure are pushed out to the 16c sites, eventually to form a rocksalt structure. The Mg insertion mechanism, “intercalation and push‐out” process, is close to the Li‐insertion mechanism in spinel oxide materials.^10^, ^11^ Then, the Mg insertion into the MgCo_2_O_4_ spinel lattice is expressed as

$${\mathrm{MgCo}}_{2}O_{4}+x\left({\mathrm{Mg}}^{\mathrm{2+}}+2e^{-}\right)↔(1-x){\mathrm{MgCo}}_{2}O_{4}+x{\mathrm{Mg}}_{2}{\mathrm{Co}}_{2}O_{4},$$

where Mg_2_Co_2_O_4_ takes a rocksalt structure, and the rocksalt structure is formed via “intercalation and push‐out” process in Figure 1, where the slight structural change or atomic rearrangement must be also accompanied by the cation insertion, which would be facilitated at moderate temperatures (about 100–150 °C). Thus, around the Mg‐inserted 16c sites, the crystal lattice undergoes the spinel‐to‐rocksalt transition, thus the atomic‐level two‐phase equilibrium can be attained, and consequently this structural change would occur coherently, as seen in Figure 1, by which deterioration of the lattice structure would be significantly suppressed.

### 2.3 Cathode Performance Tests in MRB Systems

**Figure 4:**
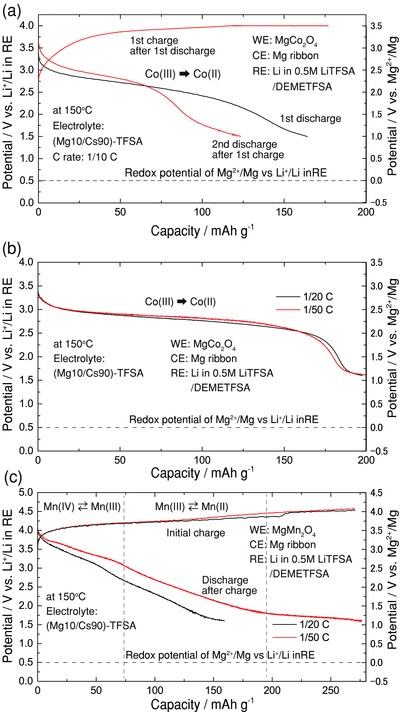
Cathode performance tests at a) 1/10 C for MgCo_2_O_4_, 1/20 C and 1/50 C for b) MgCo_2_O_4_ and c) for MgMn_2_O_4_. The ionic liquid of (Mg_10_/Cs_90_)‐TFSA (molar ratio) was used for this Mg battery system. The working electrode (cathode material) potential was plotted versus the reference electrode potential (Li metal immersed in the LiTFSA/DEMETFSA solvent).

We have successfully obtained various evidences of the Mg insertion/extraction into/from the spinel oxides. Then, let us demonstrate the high potentials of these cathode materials by constant‐current battery performance tests. In this Mg battery system using the (Mg10/Cs90)‐TFSA ionic liquid, the Mg anode is readily passivated, so that its anodic dissolution comes to occur above 1.5 V vs. Li^+^/Li in RE,^15^ which leads to an unfortunate consequence that the cell voltage decreases with the passivation of the Mg anode. Thus, the electrode potential of the cathode material (i.e., working electrode potential) was monitored versus RE. Here, referring to the CV profiles in Figure 2, we have chosen two cathode materials, MgCo_2_O_4_ and MgMn_2_O_4_ for the cathode performance tests. **Figure** **4** shows the cathode performance test for MRBs, a) 1/10 C for MgCo_2_O_4_, and 1/20 C and 1/50 C for b) MgCo_2_O_4_ and for c) MgMn_2_O_4_.

As to the cathode material MgCo_2_O_4_, the battery test can also start from the discharge process. As shown in Figure 4a, a charge process cannot be sufficiently done due to the anodic limit (oxidation decomposition around/below 3.5 V vs. Mg^2+^/Mg) of the electrochemical window of the electrolyte. Consequently, after the charge process, the discharge amount became smaller than the first one, but this is not the essential problem of the cathode material. Moreover, the cyclability is significantly affected by the thermal stability of the PVDF binder (in the compoisite active material) that cannot be endurable around 150 °C. To return to the subject, in the 1st discharge process, the converted working potential is shown to be 2–2.5 V vs. Mg^2+^/Mg, which is much higher than that of the Chevrel compounds,^8^ and the capacity amounts to about 120 mAh g^−1^ above 2 V vs. Mg^2+^/Mg and also amounts to 170 mAh g^−1^ above 1 V vs. Mg^2+^/Mg at a rate of 1/10 C.

At a slower C rate, we can obtain more excellent discharge behavior. Figure 4b,c compare the discharge (i.e., Mg insertion) behaviors of MgCo_2_O_4_ to MgMn_2_O_4_ at slower rates, 1/20 C and 1/50 C. As to MgMn_2_O_4_, the battery test was started from a charge process (over 200 mAh g^−1^ but including the oxidation decomposition of the electrolyte). In the case of 1/20 C, the MgCo_2_O_4_ cathode material shows a longer plateau region around 2.0–2.5 V vs. Mg^2+^/Mg in the potential versus capacity curve, and the discharge amount reaches about 200 mAh g^−1^. Even for a slower rate of 1/50 C, the capacity was substantially unchanged, indicating that a certain repulsive interaction associated with the push‐out process is influenced on the Mg insertion process when the cation density is increased, which is a certain kind of freezing phenomenon like glass/jamming transition.

In contrast, the MgMn_2_O_4_ cathode material displays two stages (above 2.5 V vs. Mg^2+^/Mg, 1.5–2.5 V vs. Mg^2+^/Mg) in the discharge process, which are in accordance with the redox potentials observed in the CV profile for MgMn_2_O_4_ in Figure 2d. According to the XANES spectra in Figure 3c, the higher potential region corresponds to the Mn valence change from 4 to 3, whereas the lower potential region represents the valence change from 3 to 2. It is seen that a larger capacity (about 200 mAh g^−1^) can be attained for a slower rate in MgMn_2_O_4_ (around 1.0 V vs. Mg^2+^/Mg, the influence of reduction decomposition of the TFSA anion comes to appear^15^). However, even though the two kinds of valence changes are utilized, the present test shows only about 150–200 mAh g^−1^ for MgMn_2_O_4_. As before, one of the reasons for the gradual decrease in the potential is a strain effect due to the lattice mismatch of MgO and MnO rocksalt phases, which yields a considerable strain energy depending on the insertion amount of Mg cations.^20^, ^21^ Nevertheless, the higher potential (over 2.5 V vs. Mg^2+^/Mg) of this cathode material would be a fascinating characteristics.

### 2.4 Redox Potentials by Ab Initio Calculations

Ab initio calculations were performed using GGA+*U* in order to supplement the experimental findings. Although MgCo_2_O_4_ and MgFe_2_O_4_ are disordered spinels, we considered only normal spinel configurations as it is found in our recent study^16^ that the cation configuration in MgCo_2_O_4_ does not significantly affect the redox potential caused by the Mg insertion; see Supporting Information for details. We calculated the redox potentials of Mg*X*_2_O_4_ by Mg insertion as

(1) $$V_{\mathrm{insert}}^{\mathrm{Mg}}=-\frac{1}{2e}\left[E\left({\mathrm{Mg}}_{2}X_{2}O_{4}\right)-E\left(\mathrm{Mg}X_{2}O_{4}\right)-E\left(\mathrm{Mg}\right)\right],$$

where E(A) denotes the total energy of phase *A*, *X* = Cr, Mn, Fe, or Co, and *e* is the elementary charge. The redox potential of Co_3_O_4_ by Mg insertion was also calculated as

(2) $$V_{\mathrm{insert}}^{\mathrm{Mg}}=-\frac{1}{2e}\left[E\left({\mathrm{MgCo}}_{3}O_{4}\right)-E\left({\mathrm{Co}}_{3}O_{4}\right)-E\left(\mathrm{Mg}\right)\right].$$

In each system, the most stable magnetic configuration was searched for within collinear configurations in the primitive‐based unit cell and its total energy was used in the evaluation of the redox potentials. Rocksalt ${\mathrm{Mg}}_{2}X_{2}O_{4}$ and ${\mathrm{MgCo}}_{3}O_{4}$ models were created by displacing the Mg or Co cations located at the 8a sites to neighboring 16c sites and inserting Mg cations to the remaining 16c sites. On the other hand, the redox potential of Mg extraction was calculated as

(3) $$V_{\mathrm{extract}}^{\mathrm{Mg}}=-\frac{1}{2e}\left[E\left(\mathrm{Mg}X_{2}O_{4}\right)-E\left(X_{2}O_{4}\right)-E\left(\mathrm{Mg}\right)\right].$$

The initial structures of the $X_{2}O_{4}$ phases were made by simply removing the 8a‐site Mg cations. The calculated potential vs. Mg^2+^/Mg for the insertion/extraction of Mg cations into/from the host spinel structure are summarized in **Table** **1**. The discrepancies between calculated and experimental redox potentials are within ca. 0.5 eV.

**Table 1:** Several criteria whether Mg can be inserted into the original spinel lattice or not, or whether the cations can be extracted from the original spinel lattice or not. The experimental and calculated potentials are the values vs. Mg^2+^/Mg. The judgments “Difficult” or “Feasible” are based on the present electrochemical tests within the ordinary electrochemical potential window

Spinel type	MgCo_2_O_4_ disordered	CoCo_2_O_4_ normal	MgFe_2_O_4_ disordered^19^	MgMn_2_O_4_ normal	MgCr_2_O_4_ normal
Is the counterpart phase XO of MgO in the Mg_2_X_2_O_4_ or MgX_3_O_4_ rocksalt phase stable or unstable in ambient temperature?					
Rocksalt	CoO stable	CoO stable	FeO unstablea)	MnO stable	CrO unstableb)
Is Mg_2_X_2_O_4_ or MgX_3_O_4_ rocksalt phase formed by Mg insertion via valence change from X(III) to X(II)?					
By Mg insertion	Mg_2_Co_2_O_4_ formed	MgCo_3_O_4_ formed	Mg_2_Fe_2_O_4_ partially	Mg_2_Mn_2_O_4_ formed	Mg_2_Cr_2_O_4_ not formed
Experimental CV	2.9 V	2.8 V	2.2 V	2.3 V	—
Ab initio	3.0 V	2.4 V	1.7 V	1.8 V	0.6 V
Is cation extraction possible from the host material via valence change from X(III) to X(IV)?					
By extraction	Mg_1−x_Co_2_O_4_ difficult	Co_1−x_Co_2_O_4_ difficult	Mg_1−x_Fe_2_O_4_ difficult	Mg_1−x_Mn_2_O_4_ feasible	Mg_1−x_Cr_2_O_4_ feasible
Experimental CV	—	—	—	3.4 V	3.4 V
Ab initioc)	5.3 V	—	3.5 V	2.9 V	3.9 V

^a)^FeO disproportionates to Fe and Fe_3_O_4_ in the ambient condition;^22^

^b)^CrO disproportionates to Cr and Cr_2_O_3_ in the ambient condition;^22^

^c)^The potential is calculated for the normal spinel configuration.

### 2.5 Criteria on Mg Insertion and Extraction

Here we discuss the feasibility of Mg insertion and cation extraction into/from the spinel oxides. Table 1 summarizes the experimental and calculation results and thermal stabilities of various rocksalt phases relevant to the resultant rocksalt phase. It is naturally expected that Mg_2_Co_2_O_4_ and MgCo_3_O_4_ of a random‐solution type rocksalt phase can be formed for MgCo_2_O_4_ and Co_3_O_4_, since both of MgO and CoO have similar rocksalt structures and both phases are thermally stable in the ambient condition. Similarly, since the rocksalt MnO is stable, Mg cations can easily be inserted into the MgMn_2_O_4_ host material. However, since the lattice mismatch of MnO and MgO is fairly large (as seen in the ICSD profile in Figure 3), the Mg insertion may overcome the strain energy increase. In contrast, for example, in the case of MgCr_2_O_4_ and MgFe_2_O_4_, although the MgO phase is stable, the CrO and FeO phases are less stable in the ambient condition, resultingly to disproportionate to Cr and Cr_2_O_3_ from CrO and Fe and Fe_3_O_4_ from FeO. Especially, since Cr(III) is the *d* ^3^ ion and *t*_2g_ orbitals in the octahedral crystal field are fully filled for the majority spin component, the valence change from Cr(III) to Cr(II) is quite unfavorable energetically. In such a case, the Mg insertion into the spinel host would be laborious.

On the other hand, the feasibility of Mg extraction from the original spinel oxides can basically be judged from the magnitude of the extraction potential. According to the ab initio calculation, the extraction potential is relatively high for MgCo_2_O_4_, but the potentials for MgFe_2_O_4_, MgMn_2_O_4_, and MgCr_2_O_4_ are within 3–4.5 V vs. Mg^2+^/Mg. Thus, the latter spinel oxides are expected to be demagnesiated relatively easily, and actually, among these oxides the Mg extraction tends to occur in MgMn_2_O_4_ and MgCr_2_O_4_. This is because these host materials take normal spinel structures so that the demagnesiated Mg_1−x_Mn_2_O_4_ or Mg_1−x_Cr_2_O_4_ structures would be rather robust. Since the valence change from 4 to 3 of transition metals can be utilized in this case, we can obtain a relatively higher redox potential than that by utilizing the valence change from 3 to 2. If the stability of the demagnesiated structure is ensured, one had better utilize the former valence (4 to 3) change. Furthermore, the Mg insertion with the spinel to rocksalt transition is a fascinating characteristic redox reaction. Thus, MgMn_2_O_4_ can be a high potential cathode material for MRBs in that the both redox reactions (4 to 3 and 3 to 2 in valency) can be utilized.

## 3 Conclusions

In conclusion, based on the structural similarity in spinel and rocksalt structures, we have investigated cathode properties of spinel oxides, MgCo_2_O_4_, MgMn_2_O_4_, MgFe_2_O_4_, MgCr_2_O_4_, and Co_3_O_4_, toward Mg rechargeable batteries (MRBs). In some of the spinel oxides (MgCo_2_O_4_, Co_3_O_4_, MgMn_2_O_4_), the Mg insertion and extraction can be clearly observed, which is facilitated by elevating temperature (about 150 °C). From various viewpoints, for example, electrochemistry, structural analysis, and ab initio calculation, we have substantiated the eccentric mechanism on Mg insertion into spinel‐oxide lattices, termed “intercalation & push‐out” process. This Mg insertion into a spinel structure occurs with an atomic‐level coherent phase transition, where dual‐phase reaction of the spinel and rocksalt phases proceeds. For example, in the case of spinel MgCo_2_O_4_, Mg insertion occurs at a significantly high potential of about 2.9 V vs. Mg^2+^/Mg, being consistent with ab initio calculation, and its capacity approximately amounts to 200 mAh g^−1^ (theoretically 260 mAh g^−1^). The feasibility of Mg insertion into spinel oxides would depend on the stability of both MgO and XO rocksalt phases in MgX_2_O_4_ or X_3_O_4_. In contrast, the normal spinel oxides, MgMn_2_O_4_ and MgCr_2_O_4_, can be demagnesiated to some extent, where the Mg insertion/extraction potentials of MgMn_2_O_4_ and MgCr_2_O_4_ are both about 3.4 V vs. Mg^2+^/Mg.

Since the valence change from 4 to 3 can be utilized in the latter redox reaction, a relatively higher potential is delivered, while a relatively lower potential due to the valence change from 3 to 2 is utilized in the former redox reaction. Nevertheless, even though the former redox reaction is used, since the Mg‐insertion potentials are as high as about 2–3 V vs. Mg^2+^/Mg (for MgCo_2_O_4_), the electrode energy density experimentally amounts to about 400 mWh g^−1^ (theoretically it would exceed 600 mWh g^−1^). Thus, we are sure that the “intercalation and push‐out” mechanism provides a new strategy for designing future cathode materials for polyvalent cations such as Mg cations. In addition, the spinel oxide cathode materials (e.g., MgCo_2_O_4_) can allow not only Mg‐cation insertion but also Li‐cation insertion, which enables us to design a new type of rechargeable battery, “rocking‐chair type Mg‐Li dual‐salt battery”, which is discussed in our another paper.^16^

## 4 Experimental Section

*Sample Preparation*: All spinel oxides were synthesized by the inverse co‐precipitation method.^7^, ^19^, ^23^ Aqueous metallic nitrate salt solutions (0.1 L, 0.080 m Mg(II), 0.160 m X(II), X = Co, Mn, Cr, Fe) were prepared by dissolving Mg(NO_3_)_2_·6H_2_O and X(NO_3_)_2_·*n*H_2_O, etc, in deionized water. A sodium carbonate solution (0.2 L, 0.350 m Na_2_CO_3_) for pH control and precipitation was also prepared. These solutions were heated to 70–80 °C under vigorous stirring (500 rpm). The metallic nitrate salt solutions were added dropwise into the sodium carbonate precipitation solution. The resulting suspensions were stirred at 70–80 °C for 30 min and then filtered. The filtered precipitates (precursors) were rinsed with deionized water (300 cm^3^) at 80 °C to remove completely Na‐containing by‐products, and air‐dried for 24 h at 80 °C. The precursors were followed by calcination in air at 350–750 °C for 2–24 h.

*Electrochemical Tests*: Each composite cathode was prepared by coating an Al plate collector with a mixture of the active material, carbon black (as conductive agents), and PVDF (binder) in a weight percent of 80:10:10. Mainly we used CsTFSA‐based ionic liquids containing Mg(TFSA)_2_ (and/or LiTFSA) salt established by Hagiwara et al.,^13^ where TFSA is bis(trifluoromethanesulfonyl)amide, N(CF_3_SO_2_)_2_^−^, and sometimes used an 0.5 m Mg(TFSA)_2_/triglyme electrolyte established recently.^17^ When the atomic percent of the cations in the mixed ionic liquid is, for example, Mg/Cs = 10/90, the composition of the electrolyte is denoted as (Mg10/Cs90)‐TFSA. A typical construction of beaker cells used here is illustrated in Figure 2a (upper), where a typical weight of the active materials was about 1 mg on 5 mm × 10 mm square and volume of electrolyte was about 2 ml. As a reference electrode, we used a Li ribbon instead of Mg ribbon to circumvent any passivation, which was immersed in a separated glass tube with a ceramic filter. The solvents used for the reference electrode were *N*,*N*‐diethyl‐*N*‐methyl‐*N*‐(2‐methoxyethyl)ammonium bis(trifluoromethanesulfonyl)amide (DEMETFSA) for CsTFSA‐based mixed ionic liquid and the same triglyme solvent for the 0.5 m Mg(TFSA)_2_/triglyme electrolyte, and the solution for the reference electrode was LiTFSA in both cases. As shown in Figure 2a (lower), the redox potential of Mg^2+^/Mg couple in the (Mg10/Cs90)‐TFSA ionic liquid was estimated to be about 0.5 V vs. Li^+^/Li in RE, where RE means the Li reference electrode in a glass tube separated by a ceramic filter. Moreover, the redox potential of Mg^2+^/Mg couple in the 0.5 m Mg(TFSA)_2_ in triglyme electrolyte was estimated to be about 0.8 V vs. Li^+^/Li in RE (not presented here). All the electrochemical tests and beaker‐cell construction were done with galvanostatic/potentiostatic apparatuses (Biologic, SP‐300 and VSP‐300) in the glove box whose dew point was below −72 °C.

*Structural Analysis*: The structure and valence state of the active material were investigated by X‐ray diffraction (XRD) and X‐ray absorption near edge structure (XANES), respectively, at a synchrotron radiation facility, SPring‐8. XRD patterns were acquired by the Debye‐Scherrer method with a Lindeman glass capillary and a rotating stage, where the wavelength of *λ* =1.672 Å and 0.5 Å were used. The discharged samples for the X‐ray measurements were rinsed by tetrahydrofuran (THF), dried in the glove box, followed by encapsulating into a capillary in the air atmosphere, since we confirmed that the open circuit potentials of these samples were within the electrochemical window of water. On the other hand, the charged samples were wholly prepared in the glove box by using triglyme and dimethyl carbonate instead of THF for washing to circumvent the self‐discharge of the electrode samples. The obtained capillaries were sealed by a vacuum grease and kept in an Ar atmosphere. Si powder was added to some of the samples so as to assist crushing the composite electrode before encapsulating to the capillary. The samples for the XANES measurement were prepared in the same procedure by using the same electrode samples for XRD measurements; the electrode‐shape sample with an Al collector was used for the XANES measurement. As for the charged sample, the electrode was packed by the polyimide tape to circumvent the exposure to the air atmosphere, while the bare electrode was used for the XANES measurements of discharged samples. The Rietveld analysis of XRD was performed with RIETAN‐FP.^18^ The subtraction of the background and normalization of the XANES data were carried out by using IFEFFIT.^24^ The crystal structure was drawn using VESTA 3.^25^

*Ab Initio Calculation Procedure*: To complement the experimental findings, ab initio calculations were performed using the GGA+*U* approach; the detailed procedure was described in our recent paper.^16^ The calculations were performed using the projector augmented‐wave (PAW) method^26^ as implemented in vasp.^27^ PAW data sets with radial cutoffs of 1.06, 1.32, 1.22, 1.22, 1.22, and 0.80 Å for Mg, Cr, Mn, Fe, Co, and O, respectively, were employed. Mg 3s, Cr 3d and 4s, Mn 3d and 4s, Fe 3d and 4s, Co 3d and 4s, and O 2s and 2p were described as valence electrons.^28^ We adopted Perdew‐Burke‐Ernzerhof generalized gradient approximation^29^ to density functional theory. On‐site Coulomb interactions in the 3d orbitals were corrected using the +*U* scheme proposed by Lichtenstein et al.^30^ Zhou et al. have determined effective *U* values on X‐3d (X = Mn, Fe, or Co) orbitals in various oxides using a selfconsistent scheme: 4.64–5.09, 4.90, 4.91–6.34 eV for Mn(III), Fe(III), and Co(III), respectively, which can well reproduce the voltage of Li ion batteries within a few tenth eV.^31^ Based on this report, we selected *U* = 5 eV for Mn‐ and Fe‐3d orbitals and *U* = 6 eV for Co‐3d orbitals. For Cr‐3d, we used a typical value of *U* = 5 eV. J was set at a value of 0.88 eV for all cases. Wave functions were expanded using a plane‐wave basis set with a cutoff energy of 550 eV. Spin polarization was considered for all cases. The lattice constants and internal atomic positions were fully optimized in all calculations until the residual stresses and forces converged to less than 0.24 GPa and 0.02 eV/Å, respectively.

## Acknowledgements

The authors would like to thank Ms. Chiharu Hirao for her experimental help. This work was supported by the Advanced Low Carbon Technology Research and Development Program (ALCA), Grant‐in‐Aid from the Special Coordination Funds for Promoting Science and Technology commissioned by JST, MEXT of Japan, and the MEXT Elements Strategy Initiative to Form Core Research Center. Computing resources of ACCMS at Kyoto University were used in this work. This achievement is based on the significant work on the ionic liquids by Professor Hagiwara et al. (Kyoto University) at the Research and Development Initiative for Scientific Innovation of New Generation Batteries (RISING) project from New Energy and Industrial Technology Development Organization (NEDO) of Japan.

## Supporting information

As a service to our authors and readers, this journal provides supporting information supplied by the authors. Such materials are peer reviewed and may be re‐organized for online delivery, but are not copy‐edited or typeset. Technical support issues arising from supporting information (other than missing files) should be addressed to the authors.

Supplementary
