# Supplementary material for: Intercalation and Push‐Out Process with Spinel‐to‐Rocksalt Transition on Mg Insertion into Spinel Oxides in Magnesium Batteries
Source: Adv Sci (Weinh). 2015 Jun 10;2(8):1500072. doi: 10.1002/advs.201500072 (PMC5115418; doi:10.1002/advs.201500072)
Supplement: Supplementary file 1 — Supplementary [file ADVS-2-0d-s001.pdf]

Copyright WILEY-VCH Verlag GmbH & Co. KGaA, 69469 Weinheim, Germany, 2015.

ADVANCED SCIENCE
Open Access

Supporting Information

for *Adv. Sci.*, DOI: 10.1002/advs. 201500072

Intercalation and Push-Out Process with Spinel-to-Rocksalt Transition on Mg Insertion into Spinel Oxides in Magnesium Batteries

Shinya Okamoto, Tetsu Ichitsubo, Tomoya Kawaguchi, Yu Kumagai, Fumiyasu Oba, Shunsuke Yagi, Kohei Shimokawa, Natsumi Goto, Takayuki Doi, and Eiichiro Matsubara*

Supplementary Information:
Intercalation & push-out process with spinel-to-rocksalt transition on Mg insertion into spinel oxides in magnesium batteries

Shinya Okamoto,[1] Tetsu Ichitsubo,[1*] Tomoya Kawaguchi,[1] Yu Kumagai,[2] Fumiyasu Oba,[1,2]
Shunsuke Yagi,[3] Kohei Shimokawa,[1] Natsumi Goto,[1] Takayuki Doi,[4] Eiichiro Matsubara[1]

Electrochemical stability of CsTFSA solvent

Figure S1 shows the cyclic voltammogram measured for a CsTFSA solvent (TFSA: bis(trifluoromethanesulfonyl)amide, $N(CF_3SO_2)_2{}^-$) using a Pt working electrode at 150°C. The electrodeposition and stripping of Cs is observed at around −0.2 V vs. Li^+/Li. The small cathodic peak at about 0.8 V vs. Li^+/Li corresponds to the reductive decomposition of the solvent, while the CsTFSA solvent is stable to oxidative decomposition up to about 4.5 V vs. Li^+/Li. Any anodic and cathodic peaks are not observed in the potential range of 1–4.5 V vs. Li^+/Li, suggesting that the electrochemical decomposition of the solvent is irreversible. Incidentally, the cathodic current around 1–1.5 V is considered to correspond to the cathodic decomposition of TFSA anion, as mentioned in the body text. Therefore, it can be concluded that the peak couples seen in **Figure** 2 correspond to the redox reactions of active materials.

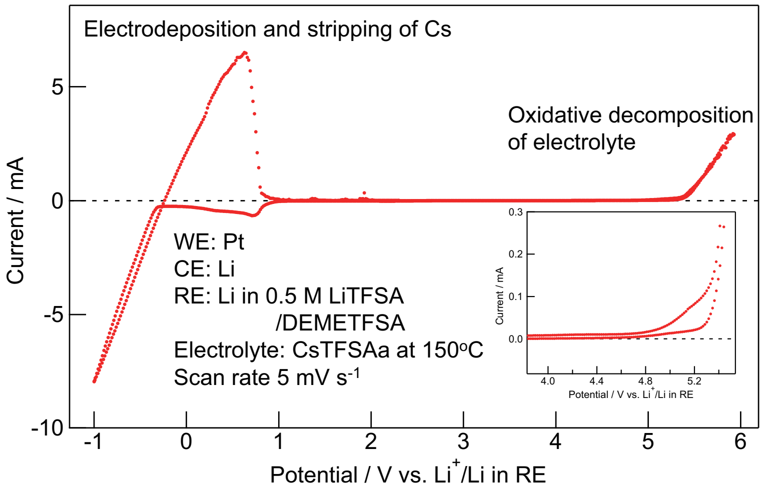

FIG. S1: Cyclic voltammogram measured in CsTFSA solvent at 150 °C.

* Corresponding: tichi@mtl.kyoto-u.ac.jp

Shape of the CV profile for the electrodeposition/stripping of Mg

In the CV profiles in **Figure** 2a, we have concluded that these redox behaviors are due to the electrodeposition/stripping of Mg. However, since we used a Mg metal as a working electrode (so as to retard the cathodic decomposition of TFSA anions), there is a possibility that Li can be inserted into Mg metal to form Mg-Li alloy. Here, we also considered this effect by using a (Li10/Cs90)-TFSA binary ionic liquid; see **Figure** S2.

The shape of the CV profile for the insertion/extraction of Li into/from the Mg matrix is crucially different from the CV shape for the electrodeposition/stripping phenomenon of Li, because the former reflects the gradual change in the chemical potential of Li (that shifts to the less-noble side), by which the current curve returning back to the anodic side overruns that toward cathodic side, leading to a less-nobler potential of the extraction of Li. This is a typical CV shape for the solid-solution phenomenon. On the other hand, in the case of the electrodeposition/stripping phenomenon, the overpotential is inevitably observed more or less on the electrodeposition, so that the current curve returning toward anodic side inevitably underruns the current curve going toward cathodic side, and it eventually crossovers with each other. Thus, the CV shapes between electrodeposition/stripping and insertion/extraction phenomena are crucially different from each other. Consequently, we have judged that the upper CV profile is due to the electrodeposition/stripping of Mg, being different from the insertion/extraction behavior of Li into/from the Mg matrix. Thus, it would be reasonable to consider that the upper CV profile is obtained due to the Mg deposition/stripping. The experimental fact that hcp Mg metal can be electrodeposited in the (Mg10/Li10/Cs80)-TFSA ternary ionic liquid[1] reasonably supports the idea. Thus, on the whole, the lithiation of Mg metal is probably also included in the CV profile more or less, but the upper CV profile mainly indicates the electrodeposition/stripping of Mg.

Rietveld analysis before/after electrochemical tests

The crystal structure parameters were determined by Rietveld refinement with the program RIETAN-FP[2] using the XRD profiles of the as-synthesized $MgCo_2O_4$ sample and one after partial discharge (120 mAh g^{-1}) in the Mg battery system that corresponds to **Figure 3a**. Although we mentioned in the body text that the rocksalt phase would have vacancies after discharge, here we assume that the discharged rocksalt structure does not have any vacancies. The cation ratio of the spinel structure was fixed at Mg/Co = 1/2, and no constraint was imposed on the cation ratio in the rocksalt structure, i.e., the discharge amount (120 mAh g^{-1}) was not taken into account for the Rietveld analysis. The fitting results and refined crystal structure parameters are shown in **Figure** S3 and Table S1, respectively. The Rietveld refinement revealed that the as-synthesized $MgCo_2O_4$ has a disordered spinel structure, and the structure of the partially discharged $Mg_{1+x}Co_2O_4$ contains spinel and rocksalt phases, whose molar fraction (or volume fraction in this case) is determined to be spinel (27%) and rocksalt (73%) structures, indicating that about 70 % of the discharge process proceeds in terms of the present structure analysis. Considering the fact that the discharge amount was less than half of the full capacity (120 mAh g^{-1}/ 260 mAh g^{-1}), we need to consider the presence of vacancies in the rocksalt crystal. Thus, the insertion of one Mg atom induces the spinel to rocksalt transition in a larger region than

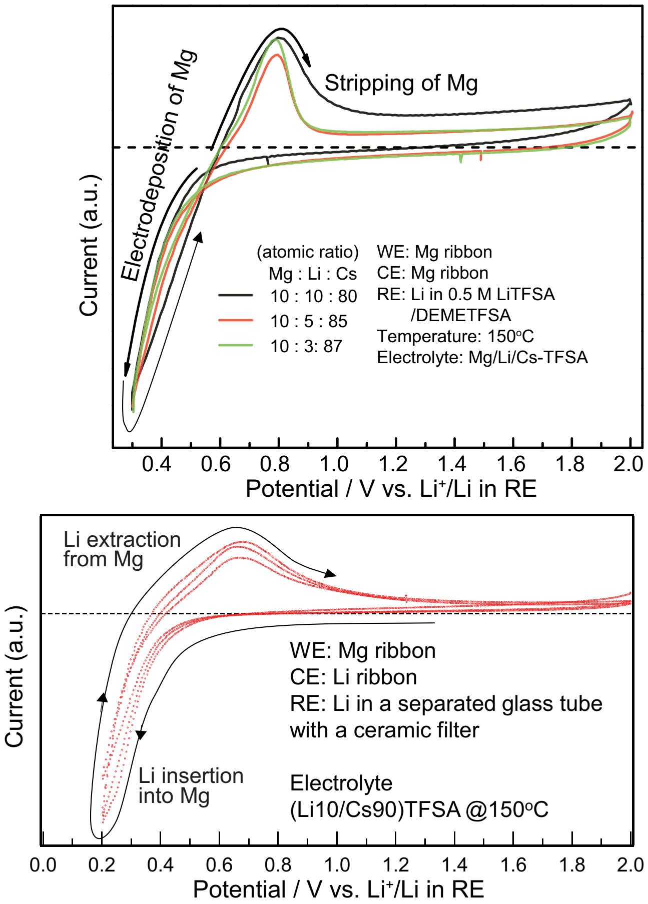

FIG. S2: The CV profiles for the electrodeposition/stripping of Mg (upper: the same as the lower panel in **Figure** 2a) and that for the insertion/extraction of Li into/from the Mg matrix (lower). The redox potential in each case is located closer, but the CV shapes are crucially different from each other.

one unit cell of the rocksalt structure.

***Ab initio* calculations**

Determination of spin and magnetic configurations in spinel $MgCo_2O_4$ We here discuss the stable spinel structure of $MgCo_2O_4$ before inserting Mg or Li cations. Since $MgCo_2O_4$ is a partially disordered spinel, the model has three degrees of freedom, i.e., the cation configuration, spin configuration, and magnetic configuration. Regarding the cation configuration, we considered all possible configurations in the primitive unit cell comprising 14 atoms, i.e., ${}_6C_2 = 15$ configurations, including symmetrically equivalent ones. For each atomic configuration, we searched for the most stable spin and magnetic configuration within the collinear magnetic configuration in the unit cell. In $MgCo_2O_4$, Co is trivalent, and can take high- or low-spin states at the tetrahedral or octahedral sites. Especially, it is well known that the energies of Co(III) high- and low-spin states at the octahedral sites, the latter of which has no local magnetic moment, are so close that both can exist depending on temperature in some materials. On the other hand, the high-spin state would be favorable for Co(III) at the tetrahedral sites. Therefore, we considered

TABLE S1: Crystal structure parameters of $MgCo_2O_4$ as-synthesized and after partial discharge (120 mAh g^{-1}) in the binary ionic liquid of $Mg(TFSA)_2$:CsTFSA=1:9 at 150°C.

Sample	Phase	mol%	a (Å)	Element	Site	g	x	y	z	B	Constraint	Rwp	S value
As-synth.	Spinel	100	8.1378(3)[a]	Mg	8a	0.571(13)	0	0	0	0.58(6)		1.331	11.408
				Co	8a	0.429	0	0	0	0.25(14)	$g = 1 - g$(Mg@8a)[b]		
				Mg	16d	0.215	0.625	0.625	0.625	0.58	$g = 0.5 - 0.5g$(Mg@8a), $B = B$(Mg@8a)[c]		
				Co	16d	0.785	0.625	0.625	0.625	0.25	$g = 0.5 + 0.5g$(Mg@8a), $B = B$(Co@8a)		
				Mg	16c	0.0199(2)	0.125	0.125	0.125	0.58	$B = B$(Mg@8a)		
				Co	16c	0.0399	0.125	0.125	0.125	0.25	$g = 2g$(Mg@16c), $B = B$(Co@8a)		
				O	32e	1	0.38507(7)	0.38507	0.38507	1.34(18)	$y = x$, $z = x$ (automatically imposed for 32e)		
Discharged	Spinel	27.35	8.156(11)	Mg	8a	0.634(4)	0	0	0	0.58		1.315	13.768
				Co	8a	0.366	0	0	0	0.25	$g = 1 - g$(Mg@8a)		
				Mg	16d	0.183	0.625	0.625	0.625	0.58	$g = 0.5 - 0.5g$(Mg@8a)		
				Co	16d	0.817	0.625	0.625	0.625	0.25	$g = 0.5 + 0.5g$(Mg@8a)		
				Mg	16c	0.032 (10)	0.125	0.125	0.125	0.58			
				Co	16c	0.063	0.125	0.125	0.125	0.25	$g = 2g$(Mg@16c)		
				O	32e	1	0.3805(3)	0.3805	0.3805	1.34	$y = x$, $z = x$ (automatically imposed for 32e)		
	Rock salt	72.65	8.5027(8)	Mg	16d	0.491	0.625	0.625	0.625	0.58	$g = g$(Mg@16c)		
				Co	16d	0.509	0.625	0.625	0.625	0.25	$g = 1 - g$(Mg@16c)		
				Mg	16c	0.491(4)	0.125	0.125	0.125	0.58			
				Co	16c	0.509	0.125	0.125	0.125	0.25	$g = 1 - g$(Mg@16c)		
				O	32e	1	0.375	0.375	0.375	1.34			

[a]The figure in parenthesis represents the error, e.g., 8.1378(3) means 8.1378±0.0003.
[b]The notation g(Mg@8a) means the occupancy of Mg at 8a site. As to the other similar notations, the same rule is applied.
[c]The notation B(Mg@8a) denotes the Debye-Waller factor for the Mg at 8a. As to the other similar notations, the same rule is applied.

both high- and low-spin states for the octahedral sites, whereas only the high-spin state for the tetrahedral sites. In total, we calculated, for each atomic configuration, 10, 11, or 12 spin and magnetic configurations without considering symmetry, which includes one non-spin state, $2^3 = 8$ magnetic configurations for the high-spin state, and 0, 1, or 2 magnetic configurations for the mixed-spin state comprising low spins for the octahedral sites. As a result, it is found that the high-spin states are more stable than the other spin states except in one structure (No. 6 in **Figure** S4), where the mixed-spin state is 0.2 eV lower in energy than the most stable high-spin state.

Mg insertion/extraction into/from spinel $MgCo_2O_4$ Next let us discuss the structures and energetics of Mg insertion/extraction into/from spinel $MgCo_2O_4$. We calculated the redox potential V of $MgCo_2O_4$ by insertion of Mg

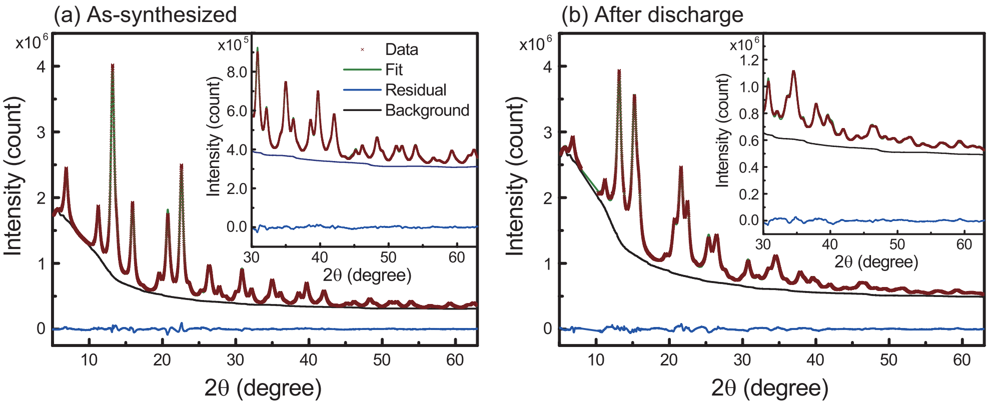

FIG. S3: Fitting results of Rietveld refinement for **a** $MgCo_2O_4$ as-synthesized and **b** after partial discharge (120 mAh g^{-1}) in the binary ionic liquid of $Mg(TFSA)_2$:CsTFSA=1:9 at 150°C.

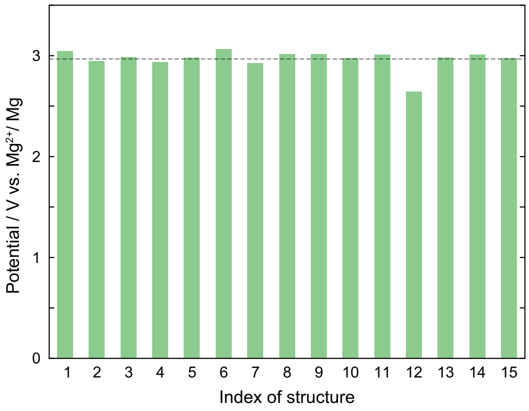

FIG. S4: Calculated redox potential of $MgCo_2O_4$ using GGA+U for all 15 possible atomic configurations in the spinel primitive unit cell by insertion of Mg to compose $Mg_2Co_2O_4$ in the rocksalt structure. The averaged redox potential is shown with a dashed line.

cations to give rise to the rocksalt structure as

$$V^{\mathrm{Mg}} = -\frac{1}{2e}[E(\mathrm{Mg_2Co_2O_4}) - E(\mathrm{MgCo_2O_4}) - E(\mathrm{Mg})],$$

where $e(> 0)$ is the elementary charge and E denote the total energies of respective phases. Mg cations were placed at the 16c sites in the primitive unit cell for each atomic configuration, and then the atomic positions and cell parameters were fully optimized. We started the structure optimization from two sets of initial atomic positions, i.e., with and without displacement of the 8a site cations into the 16c site. We considered only one cation configuration for each in the former set, since preliminary calculations indicated that the cation configuration in the rocksalt phase makes little impact on the redox potentials. Without the displacement, it has been found that some 8a cations spontaneously move to 16c sites but others do not, showing much higher energies compared to the rocksalt structures with the displacement. Subsequently, the most stable magnetic configuration was again determined for each atomic configuration. Since Co(II) is known to take the high-spin state at both of the tetrahedral and octahedral sites, we considered the high-spin state only.

Since it has been clarified that in most of configurations the total energies are substantially equal, we only considered the normal spinel structure for other spinel oxides MgX_2O_4, especially, for the extraction process. Based on the Nernst equation, we calculated the redox potential for Mg extraction

$$V^{\mathrm{ex}} = -\frac{1}{2e}[E(\mathrm{X_2O_4}) + E(\mathrm{Mg}) - E(\mathrm{MgX_2O_4})].$$

[1] M. Oishi, T. Ichitsubo, S. Okamoto, S. Toyoda, E. Matsubara, T. Nohira, R. Hagiwara, *J. Electrochem. Soc.* **2014**, 161, A943–947.

[2] F. Izumi, K. Momma, *Solid State Phenom.* **2007**, 130, 15–20.
